# Ceftazidime-avibactam resistance evolution in *Pseudomonas aeruginosa* and implications for cross-resistance to other novel β-lactams

**DOI:** 10.1128/aac.01910-25

**Published:** 2026-04-06

**Authors:** Ava J. Dorazio, Ellen G. Kline, Kevin M. Squires, Sunish Shah, Daria Van Tyne, Janet Y. Wu, Jason M. Pogue, Ryan K. Shields

**Affiliations:** 1 Department of Medicine, Division of Infectious Diseases, University of Pittsburgh, Pittsburgh, Pennsylvania, USA; 2 Antibiotic Management Program, University of Pittsburgh Medical Center, Pittsburgh, Pennsylvania, USA; 3 Center for Innovative Antimicrobial Therapy, University of Pittsburgh 6614, Pittsburgh, Pennsylvania, USA; 4 Center for Evolutionary Biology and Medicine, University of Pittsburgh 607640, Pittsburgh, Pennsylvania, USA; 5 Department of Pharmacy, Cleveland Clinic, Cleveland, Ohio, USA; 6 College of Pharmacy, University of Michigan, Ann Arbor, Michigan, USA; University of Fribourg, Fribourg, Switzerland

**Keywords:** β-lactam cross-resistance, AmpC, MexAB-OprM efflux system, ceftazidime-avibactam, ceftolozane-tazobactam, cefepime-zidebactam

## Abstract

Twelve pairs of baseline and post-exposure *Pseudomonas aeruginosa* isolates from patients treated with ceftazidime-avibactam were evaluated to define mechanisms of treatment-emergent resistance. Resistance was associated with amino acid substitutions in ampC and OXA β-lactamases, or mutations in regulatory genes conferring hyper-production of AmpC and MexAB-OprM efflux pumps. Cross-resistance was common between ceftazidime-avibactam and ceftolozane-tazobactam, less common for imipenem-relebactam and cefepime-zidebactam, and lowest for cefiderocol. These findings have important implications for sequential treatment of *P. aeruginosa* infections.

## INTRODUCTION

*Pseudomonas aeruginosa* is an opportunistic Gram-negative pathogen that frequently causes healthcare-associated infections (1). Isolates are often non-susceptible to preferred antipseudomonal β-lactams, and the organism’s intrinsic resistance mechanisms and ability to acquire resistance under antibacterial pressure make treatment-emergent resistance a persistent clinical challenge (1–3). Such adaptive trajectories can restrict subsequent therapy options for refractory *P. aeruginosa* infections, particularly when cross-resistance develops within the β-lactam/β-lactamase inhibitor (BL/BLI) class (4).

Resistance evolution during ceftolozane-tazobactam (C/T) therapy has been well described, but less is known about the mechanisms and consequences of resistance arising during ceftazidime-avibactam (CZA) treatment (5). In the multicenter CACTUS study, treatment-emergent resistance developed in 23% of CZA-treated patients, highlighting the need to understand how such evolution affects susceptibility to alternative agents (6). We recently reported that treatment-evolved resistance to cefepime-zidebactam (FEP-ZID) can confer cross-resistance to CZA and imipenem-relebactam (IPR) through antibiotic efflux-mediated mechanisms (4). This raises the question of whether CZA resistance similarly results in cross-resistance to other agents including FEP-ZID.

To investigate this question, we characterized mechanisms of cross-resistance and collateral sensitivity among multidrug-resistant (MDR) *P. aeruginosa* isolates collected from patients before and after CZA therapy. The overall objective of the study was to identify the molecular mechanisms of CZA resistance evolution and to inform effective sequential β-lactam selection in the setting of treatment-emergent resistance.

## MATERIALS AND METHODS

Baseline and post-exposure MDR *P. aeruginosa* isolates collected from patients treated with CZA were included. In each case, the baseline isolate was collected prior to CZA treatment initiation, and the post-exposure isolate was collected from the same anatomical site following treatment. Cases were selected when a ≥4-fold increase in the CZA minimum inhibitory concentration (MIC) was identified following treatment of at least 48 h within the preceding 90 days. Broth microdilution susceptibility testing was completed in triplicate according to Clinical and Laboratory Standards Institute (CLSI) guidelines with CZA, C/T, cefiderocol (FDC), imipenem (IMI), IPR, cefepime (FEP), zidebactam (ZID), and FEP-ZID (7). Avibactam, tazobactam, and relebactam were held constant at a concentration of 4 mg/L. FDC was tested using iron-depleted cation-adjusted Mueller-Hinton broth. FEP-ZID was tested in a 1:1 ratio over a range of 0.12–128 mg/L. Quality control strains *P. aeruginosa* ATCC 27853, *Klebsiella quasipneumoniae* ATCC 700603, *Klebsiella pneumoniae* BAA-1705, and *Acinetobacter baumannii* NCTC 13304 were used where appropriate, and MICs were only recorded when controls were within CLSI reference ranges (8). Across all agents, treatment-emergent resistance was defined as an MIC increase of ≥4-fold between baseline and post-exposure isolates; categorical resistance was classified according to susceptibility breakpoints established by CLSI where available (8).

Genomic DNA was extracted from isolates using a Qiagen DNeasy blood and tissue kit. Whole genome sequencing was performed using the Illumina platform (2 × 150 bp paired-end), and sequence analysis was conducted as described previously (9, 10). Mann-Whitney tests and Spearman rank correlations were used to compare MICs; visualization of data and analysis were completed using GraphPad Prism (version 10.2.3; Boston, MA).

## RESULTS

**Fig 1:**
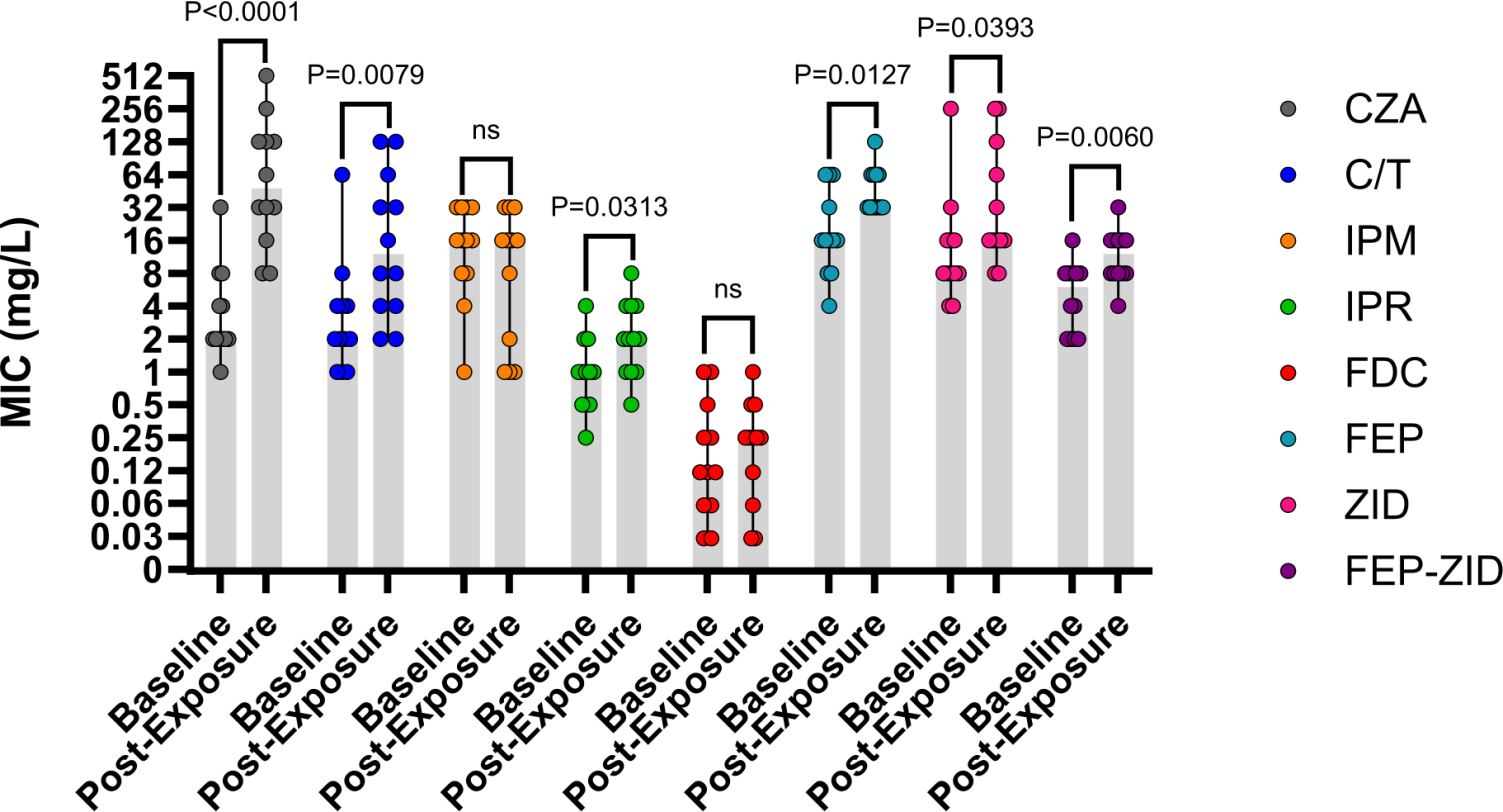
Comparison of baseline and post-exposure *P. aeruginosa* isolates collected from 12 patients treated with ceftazidime-avibactam. Notes: Median MIC values are denoted by the solid gray bars, and error bars are denoting the interquartile ranges. *P*-values were calculated by Mann-Whitney tests. C/T, ceftolozane-tazobactam; CZA. ceftazidime-avibacta; FEP, cefepime, FEP-ZID = cefepime-zidebactam; FDC, cefiderocol; IPM, imipenem; IPR, imipenem-relebactam; MIC, minimum inhibitory concentration; ZID, zidebactam.

Baseline and post-exposure paired isolates were included from 12 patients who received CZA treatment for a median (range) of 15 (10–27) days. Within patient isolates, pairs were confirmed to be the same sequence type (ST) and varied by a median of 14 (4–137) core-genome single nucleotide polymorphisms (Table S1). The median (range) baseline and post-exposure CZA MICs were 2 (1–32) and 48 mg/L (8–>256), respectively; the median fold change from baseline to post-exposure was 16-fold (4- to 64-fold). A corresponding fourfold MIC increase was identified for C/T, IPR, FDC, and FEP-ZID in 75%, 33%, 17%, and 42% of paired isolates, respectively (Fig. 1). Across patients, only one patient (Patient 5) received treatment with one of these agents (IPR for 10 days) before CZA resistance was identified (Table S1). MICs for CZA and C/T demonstrated the strongest concordance across all isolates (Spearman’s ρ = 0.76, *P* < 0.001). By categorical interpretation, 33%, 75%, and 100% of CZA post-exposure isolates demonstrated susceptibility to C/T, IPR, and FDC, respectively.

CZA treatment-emergent resistance was associated with new mutations in *ampC* (*n* = 4), *ampD* (*n* = 3), *ftsI* (PBP3; *n* = 3), *bla*OXA-10-like (*n* = 2), and efflux-associated genes, including *mexB* (*n* = 1), *mexE* (*n* = 1), *mexR* (*n* = 2), and *mexS* (*n* = 2) (Table S1). Specific AmpC substitutions included G183D (*n* = 2), Q146K (*n* = 1), and E247R (*n* = 1), and OXA-10-like substitutions included a G157D (*n* = 1) and reversion from OXA-142 to OXA-17 (D157G, *n* = 1). Median CZA MIC fold-changes were higher for isolates with mutations in *ampC* or *bla*OXA-10-like genes (*n* = 6, 48-fold) compared with those without (*n* = 6, sixfold) (*P* = 0.014). Corresponding MIC fold changes were similar for C/T (12-fold versus 4-fold; *P* = 0.067) but not for IPR (*P* = 0.41), FDC (*P* = 0.87), or FEP-ZID (*P* = 0.40). No significant MIC differences for any agents were identified between isolates with or without new mutations in efflux-related (*mexB, mexE, mexR,* mexS), *oprD,* or ampC-regulatory (*ampD, dacB*) genes.

Five of the 12 isolate pairs exhibited a ≥4-fold increase in FEP-ZID MIC from baseline. In two cases, a G183D substitution in AmpC was identified, one of which co-harbored a PBP3 substitution (R309C) that resulted in the highest FEP-ZID MIC identified (32 mg/L). In two other cases, mutations in both *ampD* and genes encoding MexAB-OprM were identified. The final pair showed a new substitution in PBP3 (R504C). Mutations in efflux-related genes were present in two of three pairs demonstrating cross-resistance to IPR, while the lone pair shows cross-resistance to FDC harbored mutations in both *ampC* and *ftsI* (encoding PBP3).

## DISCUSSION

The emergence of CZA resistance following treatment of MDR *P. aeruginosa* infections has been reported in 10%–40% of patients treated with the agent, rates that generally parallel those reported for C/T (6, 11, 12). Treatment-emergent resistance to C/T is often associated with cross-resistance to CZA and collateral sensitivity to IPR (9, 13); however, the implications of treatment-emergent resistance following CZA therapy are not yet fully understood. In this study, we found that there are at least two distinct evolutionary pathways that lead to CZA resistance. The first involves amino acid substitutions in AmpC or OXA-10-like β-lactamases, which consistently confer cross-resistance to C/T (9, 14). The second is mediated by over-production of AmpC and the MexAB-OprM efflux pump, which may confer cross-resistance to C/T and rescue treatment options like IPR or FEP-ZID. Indeed, we previously reported that mutations in genes encoding the MexAB-OprM efflux operon emerged following treatment with IPR and FEP-ZID (4, 15). The most common gene mutations identified in those studies are the same genes identified in the current analysis: *mexB* and *mexR,* which regulate efflux substrate recognition and overexpression, respectively. These data corroborate prior *in vitro* selection studies (16, 17) and underscore a growing concern that efflux-mediated resistance in *P. aeruginosa* has broad effects on β-lactam and diazabicyclooctane (DBO) β-lactamase combinations (18).

Mutations in antibiotic efflux-related genes were also the most common adaptive changes following *in vitro* selection with CZA against *P. aeruginosa* in a recent laboratory study (19). Notably, CZA exposure *in vitro* selected for cross-resistance to other β-lactams like meropenem and aztreonam, and to non-β-lactams, including ciprofloxacin and tobramycin. The latter finding has important implications for combination therapy regimens and highlights the likelihood that efflux-mediated resistance can lead to multi-class resistance. Overexpression of the MexAB-OprM operon alone does not appear to be sufficient for CZA resistance (20). Mutations in *mexA, mexB,* and *mexR* among CZA-resistant *P. aeruginosa* appear to confer resistance only when *ampC* overexpression is present, which is typically mediated by mutations in peptidoglycan recycling genes like *ampD* and *dacB*. In one recent report, a *mexR* mutation was identified in combination with a R504C substitution in PBP3 during CZA treatment of *P. aeruginosa* meningoventriculitis (21), underscoring potential complementary mechanisms of resistance involving target binding sites.

Single amino acid substitutions in the AmpC β-lactamase of *P. aeruginosa* are known to increase hydrolysis of both ceftazidime and ceftolozane (22) while impairing the enzyme’s ability to degrade imipenem (23, 24). Specifically, mutations or deletions within hot-spot regions that interact with the AmpC Ω-loop allow for a more flexible substrate binding pocket that increases the catalytic efficiency toward cephalosporins while also reducing avibactam’s inhibitory potency (22, 24). These mutations have been reported following treatment with C/T (9, 11). Our data demonstrate that the same mutations can be selected by CZA treatment, suggesting that avibactam does not prevent selection of *ampC* mutations despite its potent inhibition of wild-type AmpC β-lactamases (25), corroborating a prior *in vitro* study (24). Likewise, substitutions within the Ω-loop of OXA-2 and OXA-10-like β-lactamases have been reported following treatment with ceftazidime and C/T (26, 27). To our knowledge, this is the first clinical report of extended-spectrum OXA variants following treatment with CZA. Indeed, OXA-2 and OXA-10-like derivatives demonstrated increased C/T and CZA MICs when cloned into an isogenic *P. aeruginosa* background, and much like other serine β-lactamases, these mutations increase cephalosporin MICs at the expense of hydrolytic activity toward aztreonam, piperacillin, and meropenem (26, 28). Increased expression of OXA variant β-lactamases may also contribute to CZA resistance (27). Taken together, the data suggest that both AmpC and OXA variants conferring resistance toward C/T and CZA result in collateral sensitivity toward other β-lactams that could be used to rationally select sequential treatment options.

Our cumulative data show that the underlying mechanisms of CZA resistance in *P. aeruginosa* have critical implications to other BL/BLI combinations (4, 9, 21, 26, 29). On one hand, point mutations in β-lactamase genes confer cross-resistance to ceftazidime and ceftolozane but collateral sensitivity to aztreonam, piperacillin, and carbapenems. In this scenario, FDC and IPR are anticipated to be highly active (9, 22). We found potential evidence of cross-resistance from CZA to FEP-ZID among isolates with a G183D mutation in AmpC; however, in both cases, other mutations in resistance genes were identified (*ftsI* mutation [patient 8] and *nalC* mutation [patient 10]). The impact of AmpC substitutions selected by C/T and CZA on the *in vitro* activity of cefepime and FEP-ZID has been reported previously (30). We anticipate that such mutations will also decrease the inhibitory potency of ZID (17). Alternatively, CZA resistance can be conferred through upregulation of *ampC* and genes encoding the MexAB-OprM efflux pump, which results in varying degrees of cross-resistance to β-lactams, BL/BLI combinations, and non-β-lactam antibiotics (4, 15, 19). For example, IPR and FEP-ZID MICs increased by 1- to 16-fold and 2- to 8-fold, respectively, among post-CZA exposure isolates with mutations in efflux-related genes. Notably, no single mechanism selected by CZA treatment had a significant impact on FDC MICs in the current analysis; however, specific substitutions in AmpC and OXA β-lactamases have been reported to cause FDC resistance (28, 31). We previously found that a combination of *ampC*, *ftsI*, and *tonB* iron-dependent receptor gene mutations following treatment with C/T could lead to reduced FDC activity against MDR *P. aeruginosa* clinical isolates (29). Thus, FDC may be a viable treatment option for patients with emergent CZA resistance; however, *in vitro* susceptibility should be confirmed.

Our current investigation is limited by the relatively small number of isolates included and lack of functional validation studies, including expression of β-lactamase variants. Few patients also received other antibiotics besides ceftazidime-avibactam that may have contributed to resistance selection (Table S1). Nevertheless, these data provide a strong basis for future investigations to elucidate the specific role of each resistance mechanism identified. Our findings are strengthened by the detection of shared mechanisms across independent genetic lineages of *P. aeruginosa*. Most concerningly, we identified cross-resistance between CZA, C/T, IPR, and FEP-ZID driven by mutations in antibiotic efflux genes selected following CZA treatment. These findings support our earlier reports on IPR and FEP-ZID treatment-emergent resistance (4, 15) and highlight the vulnerability of β-lactam treatment for MDR *P. aeruginosa* in clinical practice. Future studies are needed to define strategies to suppress resistance selection and leverage collateral sensitivity pathways to optimize clinical management for this notoriously difficult-to-treat pathogen.

## SUPPLEMENTAL MATERIAL

Table S1 aac.01910-25-s0001.xlsx Phenotypic and genotypic characteristics of MDR *P. aeruginosa* isolates collected before and after patient treatment with ceftazidime-avibactam.

ASM does not own the copyrights to Supplemental Material that may be linked to, or accessed through, an article. The authors have granted ASM a non-exclusive, world-wide license to publish the Supplemental Material files. Please contact the corresponding author directly for reuse.
